# Annotations

**Published:** 1887-10-08

**Authors:** 


### Annotations.

Coroners: Legal or Medical?

In this world birds with the longest beaks and the sharpest claws generally get the biggest share of the carcase. If a man cannot take care of himself—woe be to him! It is the same with a profession. Doctors belong to domestic rather than to public life, and they acquire habits of timidity and unreadiness, which to a large extent unfit them for conflict with their fellow-men. Besides, it cannot be denied that many of them have foolish and purely conventional notions about professional dignity. There is nothing undignified about self-defence; nor indeed about aggressive warfare, if the cause be just and such as to demand aggression. Lawyers, on the other hand, are as keen as hawks, and quite as hungry. If there be any pickings going they will not be very far off. All is fish which comes to their net; and hence they often usurp the purely medical function of holding an inquiry as to the cause of death. No coroner's appointment is ever vacant, but some wide-awake barrister or solicitor will lay his claim to it. This is absurd. The coroner sits to hold an inquest into the cause of death. That is his primary and practically his sole function: the very essence of his duty. The inquiry as to motives influencing the dead or any other person connected with the death, is quite subsidiary. But even if it were not, the medical man is much better fitted both by education and experience to weigh and adjudicate upon the kind of evidence given in coroners' courts than the lawyer. Dr. Vacher, Medical Officer of Health for Liverpool, in a paper read at the recent Sanitary Congress at Bolton, strongly condemns the system of legal coroners; and every instructed person of whatever calling cannot but entirely agree with him. For this, as for other humiliations, doctors have themselves principally to thank.

Miss Twining on Village Life.

Miss Louisa Twining endeavours to dispel some of the illusions prevalent about the life of the poor in villages. It is popularly supposed, or at least it used to be before the Rev. Dr. Jessop took to instructing and amusing the public in the pages of the *Nineteenth Century*, that the village poor lived a life not only of ideal simplicity, but of robust healthiness and perpetual youth. The "Isaac Ashfords" and "Village Hampdens" were credited with brawny muscles, cheeks that matched the roses in the richness of their bloom, and virtues untainted by any contact with towns. Dr. Jessop has demolished the "village virtues" theory, and now comes Miss Twining to dispel the visions of muscles of iron and peach-coloured complexions. During her summer wanderings in recent years she has been more and more struck by the number of pale faces in country towns and villages. She does not doubt that the natural conditions of life in these places are healthy, but these conditions are rendered nugatory chiefly by the ignorance of the poor, and even of the middle-classes, as to all knowledge of the laws of health and their prejudice against enlightenment. The three chief causes are want of air in ventilation, want of water in ablution, and want of wholesome food through ignorance of cookery, even of the simplest description. There is some truth in what Miss Twining says, but it is very probable that her researches have been confined to southern villages, especially in what are called the "home counties." Had she gone farther afield, as for example to the agricultural districts of North Lincolnshire and Yorkshire, where the labourers still feed at the farmer's table, and on food of his providing, she would have seen men and lads that match the horses at the plough in healthful bloom and sturdiness of build. Miss Twining's observations are confined within too narrow a range. Her diagnosis of the disease may be fairly correct, but her view of the causes does not go deep enough. At the root of the helplessness and ignorance of the poor in those southern counties, where they are less prosperous than elsewhere, lie ages of hereditary feebleness, induced by small wages, poor food, and the absence of all hope. Their ignorance of cookery arises from the fact that they have never been able to buy anything to cook: and their indifference to ventilation is the result of living in houses in which no provision for ventilation has ever been made. A hundred years ago the highest and wealthiest knew nothing of ventilation. The poor are simply a hundred years behind in knowledge, and a thousand years in resources and hope. If Miss Twining, or some other well-disposed philanthropist, would do as one of Mr. Walter Besant's heroines did, and for a single year divest herself of her wealth and live in a cottage with labourers; eating, sleeping, dressing, planning, contriving, fearing, and suffering every kind of disheartening defeat which falls to the lot of those whose resources bear no just relation to their labours and responsibilities, she would be able, like a physician of profound skill, to put her finger on the true causes of the evil, and so to point the way to its complete and permanent cure.

Ladies, Physic, and Mr. Ruskin.

We have never been of that numerous company who think ladies unfit for the study and practice of physic. It is one of the shallowest of shallow philosophies which would prevent women studying and practising any honest art for which they have the capacity and the opportunity; and the only way of testing the capacity is by giving needful facilities for its exercise. Abstract reasoning is one method of aiming at truth, but repeated experiment is another and a very much better way. If Mr. Smith thinks he has made a pair of wings which will bear him safely through the air, by all means give him the keys of the parish church, and let him try a preliminary flight from the steeple. He will soon know the real value of his wings, and so will his neighbours. Mrs. Scharlieb, M.B. and B.S., has just addressed the lady students of medicine at the opening of the winter session of their medical school in Henrietta Street. The subject she chose well illustrates the advantages which may arise to the profession of medicine by the admission of women to its ranks. It was a subject which no medical *man* would have publicly dealt with, lest he should be thought imaginative and unpractical. The average practitioner is terribly narrow in some directions. There is a kind of unwritten system of professional philosophy which dictates for him a severely practical and unimaginative line of things, beyond which he may not stray lest he be classed with the Hahnemanns and Browns of unscientific and doubtfully honest notoriety. Mrs. Scharlieb discoursed on the "Seven Lamps of Medicine," taking her lead and inspiration from Mr. Ruskin's "Seven Lamps of Architecture." And why not, pray? Is there absolutely no room whatever for the figurative and the poetical in the science and art of physic?

